# Antibiotic prophylaxis in vesicoureteral reflux: A paradigm shift

**Published:** 2009

**Authors:** J. S. Banerji, J. C. Singh

**Affiliations:** Department of Urology, Christian Medical College, Vellore, India E-mail: chandrasingh@cmcvellore.ac.in

## SUMMARY

In this prospective trial, Pennesi *et al,*[1] randomized 100 children with vesicoureteral reflux (VUR) into a treatment arm, which received two years of oral sulfamethoxazole/trimethoprim and a control arm that did not receive any chemoprophylaxis. Based on prior trials, a sample size of 48 was required for a 30% absolute risk difference. Analysis was done on an intention to treat basis. Children between the age of 1 day and 30 months at first episode of pyelonephritis were included. As Grade 1 would spontaneously resolve in the majority, and grade five has a high probability of being associated with renal dysplasia, only children who had grades 2-4 VUR were included. Voiding cystourethrography was performed in all, two months following the acute pyelonephritis. Renal ultrasound and DMSA scan were performed at six months. Although antibiotic prophylaxis was discontinued at two years in the intervention group, all subjects were followed up for a total period of four years. The results were quite an eye opener. Contrary to prior understanding, in the chemoprophylaxis group, there was a slightly higher risk, though not statistically significant, for developing pyelonephritis (RR 1.42 in the first year and 1.25 in the second year). 36% children in the prophylaxis group and 30% in the control group had at least one episode of pyelonephritis in the first two years. In the subsequent two years, both groups did not receive any prophylaxis. Only one in the intervention group and two in the control group developed pyelonephritis. However, it is noteworthy that all pyelonephritis episodes in the intervention arm were caused by multiresistant bacteriae, as opposed to sensitive organisms in the control group. There was no difference in the rate of renal scars or resolution of reflux between both groups. The authors conclude that antibiotic prophylaxis is ineffective either to prevent pyelonephritis recurrence, or to alter the course of the primary pathology.

## COMMENTS

The cornerstone of management of VUR was hitherto thought to be antibiotic prophylaxis, on the premise that upper tract infection and renal damage could be prevented. Although we know from Hodson's water hammer theory that Scars could occur in sterile reflux, Edwards and Smellie[2] in their landmark publication in 1977 concluded that antibiotic prophylaxis had come to stay. Though surgical intervention has been proven to be ineffective to prevent progression of renal deterioration,[3] none of the trials had a control group without any antimicrobial prophylaxis. Several attempts were made to evaluate the effectiveness of antibiotic therapy in reducing incidence of recurring infections. But the evidence had been scant and of poor quality. Against this backdrop, the effort of the authors to study the role of chemoprophylaxis is commendable. Though the study was not blinded, the radiologists interpreting the DMSA scans and the operator analyzing the urine cultures were blinded. As the criteria are stringent and the parameters studied are objective, the likelihood of any bias is minimal. In a cohort study by Conway *et al,*[4] involving 611 children who had first episode of urinary tract infection (UTI), antimicrobial prophylaxis was not associated with decreased risk of recurrent UTI (HR, 1.01; 95% CI, 0.50-2.02), but was a risk factor for antibimicrobial resistance among children with recurrent UTI (HR, 7.50; 95% CI, 1.60-35.17). Similar conclusions have been drawn By Garin *et al,*[5] wherein 218 children with pyelonephritis were randomized to receive antibiotic prophylaxis or not. The distribution of reflux was similar. Follow up after one year revealed that there were no statistically significant differences among the groups with respect to rate or type of UTI recurrence or development of renal parenchymal scars. The scales seem to have tilted towards management of VUR without antibiotic prophylaxis. There would still be a small sub group of girls with recurrent pyelonephritis, who would need antibiotics alone or in conjunction with surgery, but by and large the writing on the wall appears fairly clear that prophylaxis does not change the course of a majority of vesicoureteric reflux.
